# Subcutaneous adipose tissue fatty acid desaturation in adults with and without rare adipose disorders

**DOI:** 10.1186/1476-511X-11-19

**Published:** 2012-02-03

**Authors:** Jennifer K Yee, Susan A Phillips, Kambiz Allamehzadeh, Karen L Herbst

**Affiliations:** 1 Los Angeles Biomedical Research Institute at Harbor-UCLA Medical Center, Department of Pediatrics, Division of Endocrinology, 1000 West Carson Street, Harbor Box 446, Torrance, CA 90509, USA; 2 University of California, San Diego and Rady Children's Hospital San Diego, Department of Pediatrics, Division of Endocrinology, Veteran's Affairs San Diego Health Care System, 3350 La Jolla Village Drive, San Diego, CA 92161, USA; 3 Veteren's Affairs San Diego Health Care System, Mailcode 9-111-G, 3350 La Jolla Village Drive, San Diego, California 92161, USA; 4 University of California, San Diego, Department of Internal Medicine, Division of Endocrinology, Veteren's Affairs San Diego Health Care System, Mailcode 9-111-G, 3350 La Jolla Village Drive, San Diego, California 92161, USA

**Keywords:** Adipose tissue, Gas chromatography, Fatty acid, Desaturation, Lipomatosis, Obesity

## Abstract

**Background:**

Elevated stearoyl-CoA desaturase activity has been described in obese states, with an increased desaturation index (DI) suggesting enhanced lipogenesis. Differences in the DI among various phenotypes of abnormal adiposity have not been studied. Abnormal accumulation of subcutaneous adipose tissue occurs in rare adipose disorders (RADs) including Dercum's disease (DD), multiple symmetric lipomatosis (MSL), and familial multiple lipomatosis (FML). Examining the DI in subcutaneous fat of people with DD, MSL and FML may provide information on adipose tissue fatty acid metabolism in these disorders. The aims of this pilot study were: 1) to determine if differences in adipose tissue DIs are present among RADs, and 2) to determine if the DIs correlate to clinical or biochemical parameters.

**Methods:**

Subcutaneous adipose tissue was obtained from human participants with DD (n = 6), MSL (n = 5), FML (n = 8) and obese Controls (n = 6). Fatty acid composition was determined by gas chromatography/mass spectrometry. The DIs (palmitoleic/palmitic, oleic/stearic, vaccenic/stearic ratios) were calculated from the gas chromatogram peak intensities. SCD1 gene expression was determined. Spearman's correlations between the DIs and available clinical or biochemical data were performed.

**Results:**

In DD subjects, the vaccenic/stearic index was lower (*p *< 0.05) in comparison to Controls. Percent of total of the saturated fatty acid myristic acid was higher in DD compared with Controls and FML. Percent of monounsaturated vaccenic acid in DD trended lower when compared with Controls, and was decreased in comparison to FML. In MSL, total percent of the polyunsaturated fatty acids was significantly lower than in the Control group (*p *< 0.05). In the total cohort of subjects, the palmitoleic/palmitic and oleic/stearic DIs positively correlated with age, BMI, and percent body fat.

**Conclusions:**

The positive associations between the DIs and measures of adiposity (BMI and percent body fat) support increased desaturase activity in obesity. The lower vaccenic/stearic DI in DD SAT compared with Controls suggests presence of other factors involved in fat accumulation in addition to lifestyle. Other mechanisms driving fat accumulation in DD such as inflammation or lymphatic dysfunction should be investigated.

## Background

Altered fatty acid metabolism has been implicated in the development of obesity. Elevated fatty acid desaturase activity by determination of the desaturation index is being studied as a potential biomarker of metabolic risk. Saturated fatty acids (SFA) made by de novo synthesis or taken up from the diet are converted to delta-9 monounsaturated fatty acids (MUFA) by stearoyl-CoA desaturase enzyme 1 (SCD1), a membrane-bound enzyme highly expressed in the liver and adipose tissue. The desaturation index (DI) is the ratio of MUFA product to SFA precursor, and is a measure of SCD1 activity since it has been shown to correlate with SCD1 gene expression, protein expression, and enzyme activity [1,2].

Decreased SCD1 activity has been reported in the proinflammatory settings of Crohn's disease and coronary artery disease [3-5]. Increased SCD1 activity has been demonstrated in obese states [2,6,7] with correlation to other markers of abnormal metabolism [8]. However, whether variations in desaturase activity exist among different obese phenotypes has not been sufficiently investigated, and we applied this question to the study of human subjects with rare adipose disorders (RADs) (Table 1).

**Table 1:** Rare adipose disorders and their clinical features

Disorder	Synonym	Pattern of Abnormal SAT	Clinical Features
Dercum's disease (DD) [9] Type I Type II Type III	Adiposis dolorosa Morbus Dercums	Type I: Juxta-articular Type II: Diffuse, associated with obesity Type III: Discrete, fatty lumps	Painful subcutaneous adipose tissue in all types Male-female ratio 1:5-30 Fatigue, myalgias, shortness of breath [10] Autosomal dominant transmission has been reported [11]

Familial multiple lipomatosis (FML)	None	Discrete, lipomas +/- encapsulation; arm, thighs, abdomen, lower back, flanks [12,13]	Non-painful. Male-female ratio 1:1 If lipomatosis becomes painful, is considered DD Type III [11,14,15]. Autosomal dominant transmission; recessive inheritance has been reported.

Multiple symmetric lipomatosis (MSL)	Lanois-Bensaude's syndrome Benign symmetric lipomatosis Madelung's disease	Diffusely in men on neck and back, upper arms in women; supraclavicular, on abdomen, and lower back in both men and women [16]	Non-painful. Male-female ratio 15:1-30:1 Polyneuropathy [17] Mitochondrial mutations found in some [18] Risk factors: corticosteroids and alcohol abuse.

RADs involve the abnormal formation of fatty masses in the subcutaneous adipose tissue (SAT). RADs include Dercum's disease (DD), multiple symmetric lipomatosis (MSL) and familial multiple lipomatosis (FML). These disorders differ in whether or not pain is present and in the location of fatty masses [10,16,19]. The pathophysiology of RADs is believed to be different from the generalized accumulation of fat in obesity because the abnormal SAT follows discrete patterns, can occur in both obese and non-obese individuals [10,20], may include loss of normal fat (as in MSL)[21], and may involve alterations of the lymphatic system [21,22]. A previous study suggested that inflammation is involved in the selective expansion of the adipose tissue in DD through elevated interleukin IL-6 expression in affected SAT, and increased blood IL-13 levels in DD women compared to controls [23]. However, another study found no difference in the number of perivascular immune cells in the painful SAT of women with DD in comparison to the non-painful SAT of a subset of these women, and in comparison to the SAT samples from healthy, pain-free, obese and normal weight control subjects [24].

Previous data on fatty acid analyses from DD SAT compared to normal adipose tissue is limited. However, available data suggests that the formation of 18-carbon fatty acids, particularly desaturation of stearic (18:0) to oleic (18:1) acid may be blocked in DD (decreased desaturation) [25]. More recent work, however, suggests an elevation in MUFA in the DD SAT (increased desaturation) [26] compared to non-obese controls. There is little available data on fatty acid composition in FML and MSL. However, one study suggested elevated monounsaturated palmitoleic acid in SAT of MSL subjects [27].

The objectives of this study were 1) to determine if differences in adipose tissue DIs are present among various phenotypes of obesity, and 2) to determine if the adipose tissue DIs correlate to biochemical or clinical parameters. We hypothesized that differences in the fatty acid profiles and DIs exist between the RAD groups and obese controls. We also hypothesized that the DIs correlate to clinical measures of adiposity, and that the correlation patterns may differ between groups. We determined the fatty acid profiles and desaturation indices in DD, FML, and MSL in comparison to obese Controls using gas chromatography/mass spectrometry (GC/MS), and examined correlation patterns between the desaturation indices and biochemical or clinical parameters.

## Results

### Subjects

Subject demographics are presented in Table 2. There were no significant differences between groups for age or body mass index (BMI). There were more women in the DD and MSL groups and more men in the FML group compared to Controls.

**Table 2:** Subject demographics, and BMI.

	Group			
	**Control (n = 6)**	**DD (n = 6)**	**FML (n = 8)**	**MSL (n = 5)**

Age (years)	49.0 (42.0-58.0)	46.5 (40.0-52.0)	38.5 (37.5-46.5)	50.0 (40.3-50.3)

Males/females (n)	4M/2F	2M/4F	6M/2F	1M/4F

BMI (kg/m^2^)	34.7 (32.2-38.1)	29.2 (28.0-35.0)	30.5 (27.3-32.0)	32.1 (31.8-46.5)

The median is presented with the 25-75% interquartile ranges

*DD *Dercum's disease, *FML *Familial multiple lipomatosis, *MSL *Multiple symmetric lipomatosis

### Fatty acids

Table 3 lists the fatty acids in the order of elution on the gas chromatogram. Nine major chromatogram peaks, each representing a different fatty acid, were observed in all samples from RAD and Control subjects, except for arachidonic acid, which was not detected in one DD subject and one FML subject. These nine peaks corresponded to myristic, palmitic, palmitoleic, stearic, oleic, vaccenic, linoleic, α-linolenic, and arachidonic acids. Three additional peaks (designated by ¥) were detectable in less than half of the subjects so comparisons were not made between groups, but are included in Table 3 for interest. Two of these peaks are referred to as 18:1X and 18:1Y, due to the general inability to determine position of the double bond by GC/MS, and the third peak was thought to be eicosenoic acid. Eicosapentaenoic acid (EPA, 22:5) and docosahexaenoic acid (DHA, 22:6) were undetectable in the study subjects.

**Table 3:** Subject fatty acid profiles.

	Group			
	**Control (n = 6)**	**DD (n = 6)**	**FML (n = 8)**	**MSL (n = 5)**

	**Saturated and Monounsaturated Fatty Acids Percent of Total (SEM)**			

14:0 (myristic)	1.22 (1.17-1.38)	1.74 (1.52-1.78)**†	1.30 (1.08-1.47)	1.53 (1.12-2.29)

16:0 (palmitic)	27.4 (24.8-28.5)	27.3 (26.0-29.2)	25.4 (25.2-28.3)	26.3 (23.0-31.6)

16:1n-7 (palmitoleic)	3.68 (2.90-4.04)	2.78 (2.18-3.43)	2.71 (2.27-3.28)	4.04 (3.24-4.95)

18:0 (stearic)	4.15 (3.93-5.52)	6.07 (5.81-7.92)	5.80 (5.59-7.21)	3.88 (2.21-7.72)

18:1X ^¥^	1.95 (1.87-2.54)	1.52 (1.12-1.92)	1.91 (1.43-2.01)	1.71 (1.27-2.14)

18:1n-9 (oleic)	47.8 (46.1-48.9)	48.5 (47.5-49.0)	48.2 (46.9-48.7)	46.6 (43.3-51.9)

18:1n-7 (vaccenic)	3.22 (2.72-5.70)	2.01 (1.72-2.61)**†‡**	5.85 (4.09-6.25)	2.95 (2.64-6.31)

18:1Y ^¥^	0.82 (0.75-0.94)	not detected	2.22 (2.05-2.38)	0.82 (N/A)

20:1n-9 (eicosenoic) ^¥^	0.65 (0.60-0.73)	0.60 (0.52-0.72)	0.70 (0.62-0.79)	0.50 (0.49-0.54)

Total percent SFA	33.4 (29.1-34.8)	36.2 (35.7-38.6)	33.0 (31.1-35.5)	31.3 (26.6-41.7)

Total percent MUFA	57.9 (54.8-59.8)	54.4 (53.5-56.2)	57.0 (55.2-60.2)	56.4 (53.1-61.0)

	Polyunsaturated Fatty Acids Percent of Total (SEM)			

18:2n-6 (linoleic, LA)	9.12 (8.31-10.4)	8.34 (8.10-8.89)	9.65 (8.07-9.94)	5.64 (4.61-8.50)**‡**

18:3n-3 (α-linolenic, ALA)	0.23 (0.17-0.26)	0.24 (0.18-0.32)	0.21 (0.17-0.26)	0.15 (0.12-0.17)**‡†**

20:4n-6 (arachidonic, AA)	0.13 (0.10-0.16)	0.10 (0.09-0.13)	0.13 (0.11-0.20)	0.14 (0.13-0.41)**^#^**

Total percent PUFA (LA+ALA+AA)	9.49 (8.58-10.8)	8.61 (8.31-9.29)	10.0 (8.35-10.3)	6.21 (5.07-8.80)*

n-6/n-3 ratio (LA+AA)/ALA (arbitrary units)	43.3 (40.1-50.4)	36.8 (29.2-47.1)	44.6 (37.7-51.4)	40.1(30.5-50.3)

The median percent of total is presented with the 25-75% interquartile range in parentheses. In DD, myristic acid percent of total is increased compared to Controls and FML. Vaccenic acid percent of total trends toward a decrease compared to Controls, but is significantly lower compared to FML

*DD *Dercum's disease, *FML *Familial multiple lipomatosis, *MSL *Multiple symmetric lipomatosis

*p < 0.05 vs. Control; **p < 0.01 vs. Control; †*p *< 0.05 vs. FML; ‡ trended lower vs. Control (see text);

^#^trended higher vs.DD; ^¥^no comparison between groups made due to low prevalence in the groups; *N/A *non-applicable because not detected in enough subjects to provide a value

The saturated myristic acid percent of total was significantly higher in DD than in the Control (*p *< 0.01) and FML groups (*p *< 0.05). The monounsaturated vaccenic acid percent of total in DD trended lower than Controls, and was significantly decreased compared to FML (*p *< 0.05). The abundance of linoleic acid (LA) trended lower in MSL versus the Control (*p *= 0.05) and FML groups. The α-linolenic acid (ALA) percent of total in MSL trended lower in comparison to Controls (*p *= 0.05), but was significantly lower when compared to FML (*p *< 0.05). Arachidonic acid (AA) trended higher in the MSL group compared with DD. The sum percent of total of the detected PUFA was significantly lower in MSL (*p *< 0.05), and trended lower in DD, compared with Controls.

### Desaturation and elongation indices

The oleic/stearic DI in the DD group trended lower versus the Control group (Table 4). The DI for vaccenic/stearic was significantly lower in the DD group compared to Controls (*p *< 0.05) and when compared to the FML group (*p *< 0.01). There was no difference in the palmitoleic/palmitic DI among the groups. The (palmitoleic + vaccenic)/palmitic DI for the DD group trended lower compared to the Control group, and was decreased in comparison to the FML group (*p *< 0.05).

**Table 4:** The desaturation indices and the elongation index.

Index	Group			
	
	Control (n = 6)	DD (n = 6)	FML (n = 8)	MSL (n = 5)
Desaturation Index				

palmitoleic/palmitic	0.13 (0.12-0.14)	0.09 (0.08-0.14)	0.11 (0.08-0.13)	0.13 (0.11-0.25)

oleic/stearic	11.4 (8.5-12.4)	7.8 (6.2-8.5)**‡**	8.4 (6.5-9.7)	13.2 (5.8-21.1)

vaccenic/stearic	0.86 (0.66-1.34)	0.33 (0.22-0.46)*†	0.81 (0.72-1.37)	0.58 (0.38-3.26)

(palmitoleic+vaccenic)/palmitic	0.27(0.23-0.34)	0.18 (0.15-0.21)**†‡**	0.34 (0.27-0.36)	0.37 (0.20-0.43)

Elongation Index				

stearic/palmitic	0.18 (0.15-0.25)	0.22 (0.20-0.30)	0.24 (0.19-0.27)	0.15 (0.10-0.24)

Fatty acid ratios are presented as the median in arbitrary units with the 25-75% interquartile range in parentheses. DD demonstrates a decrease in the vaccenic/stearic ratio in comparison to Controls and FML

*DD *Dercum's disease, *FML *Familial multiple lipomatosis, *MSL *Multiple symmetric lipomatosis

*p < 0.05 vs. Control; †*p *< 0.05 vs. FML; ‡ trended lower vs. Control (see text)

There were no differences among the groups for the stearic/palmitic elongation index.

### Adipose tissue expression of SCD-1

The RNA was of good quality and the cDNA performed well when tested with reference genes (data not shown). SCD-1 gene expression was below the limits of detection in all groups, including obese Control subjects (data not shown).

### Correlations between fatty acid indices and clinical or biochemical parameters

**Figure 1:**
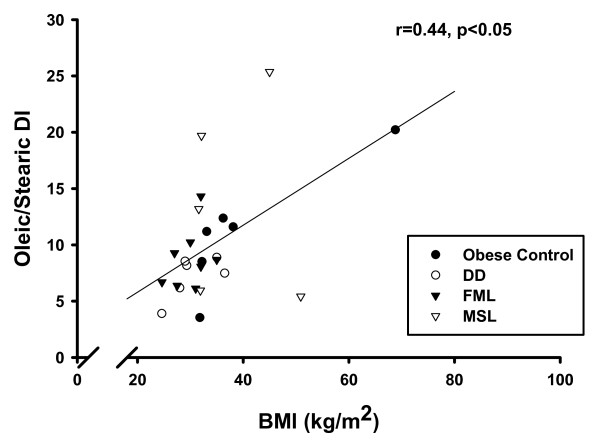
**BMI versus the oleic/stearic DI in the total cohort of subjects**. There was a positive correlation between BMI and the oleic/stearic DI, r = 0.44, *p *< 0.05.

**Figure 2:**
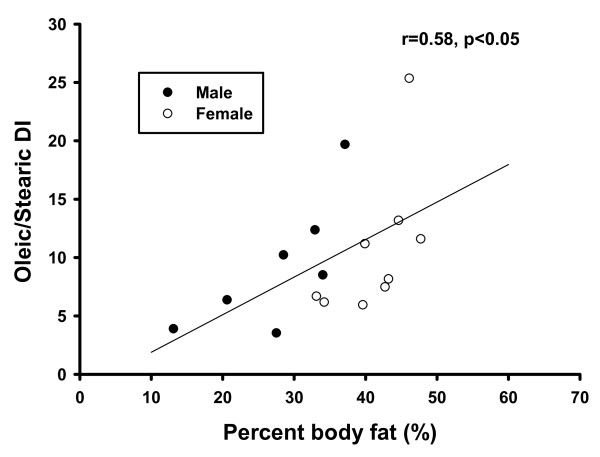
**Percent body fat versus the oleic/stearic DI in the total cohort of subjects**. There was a positive correlation between percent body fat and the oleic/stearic DI, r = 0.58, *p *< 0.05. This association was present in both males and females.

Adipose tissue samples were obtained from two different studies, therefore the biochemical data and measurements were incomplete. The data and number of subjects for each measure are presented in Table 5. In the total cohort of subjects, the palmitoleic/palmitic, oleic/stearic, vaccenic/stearic, (palmitoleic + vaccenic)/palmitic DI all correlated positively with each other, and negatively with the stearic/palmitic ratio. Age positively correlated with palmitoleic/palmitic (r = 0.51, *p *< 0.05) and the oleic/stearic DI (r = 0.48, *p *< 0.05). BMI also positively correlated with the palmitoleic/palmitic (r = 0.40, *p *< 0.05) and the oleic/stearic DI (r = 0.44, *p *< 0.05) (Figure 1), but was negatively correlated with the stearic/palmitic ratio (r = -0.59, *p *< 0.01). Percent body fat was positively associated with the palmitoleic/palmitic (r = 0.51, *p *< 0.05) and oleic/stearic DI (r = 0.58, *p *< 0.05, Figure 2), and negatively associated with the stearic/palmitic ratio (r = -0.57, *p *< 0.05). Among females, percent body fat correlated with the oleic/stearic DI (r = 0.83, *p *< 0.01). Among males, percent body fat correlated with both the palmitoleic/palmitic DI (r = 0.86, *p *< 0.01) and the oleic/stearic DI (r = 0.79, *p *< 0.05). A correlation pattern could not be confirmed within RAD and Control groups due to small subject numbers.

**Table 5:** Clinical and biochemical parameters in the total cohort.

Clinical or biochemical parameter (units, number of subjects)	Median value (25-75% interquartile range)
Age (years, n = 25)	47 (39-51)

BMI (kg/m^2^, n = 25)	32.0 (29.2-35.3)

Percent body fat (%, n = 16)	35.7 (30.7-43.0)

Total cholesterol (mg/dL, n = 22)	188 (174-201)

LDL (mg/dL, n = 19)	114 (100-132)

HDL (mg/dL, n = 19)	53 (38-56)

Triglycerides (mg/dL, n = 22)	122 (98-160)

Leptin (ng/mL, n = 9)	13.9 (4.9-32.7)

Adiponectin (μg/mL, n = 9)	27.4 (12.5-32.2)

Non-esterified fatty acids (mmol/L, n = 14)	0.53 (0.38-0.75)

The values are presented as the median with the 25-75% interquartile range

The oleic/stearic, vaccenic/stearic, and (palmitoleic + vaccenic)/palmitic acid DI correlated negatively with adiponectin (r = -0.82, *p *< 0.01; r = -0.80, *p *< 0.01; r = -0.78, *p *< 0.01 respectively) while the stearic/palmitic elongation index correlated positively (r = 0.88, *p *< 0.001). No associations were found between leptin and the DI.

## Discussion

For the first time, the fatty acid composition and desaturation indices are presented in three RADs compared with obese Controls. We hypothesized that fatty acid composition may be different if the SAT developed in a person with a RAD, with the fatty acid profile reflecting changes in desaturase activity. Fatty acid DIs reflect the proportion of MUFA product generated from the SFA precursor and is known to be upregulated in models of obesity, indicating enhanced lipogenesis [2,7,28]. We demonstrate in this study that the vaccenic/stearic DI in SAT from participants with DD, was lower than that of obese Controls, suggesting that the SAT in DD is a biochemically distinct subtype of SAT.

A contributing factor to the differences in the DIs was a trend to decreased vaccenic acid percent of total in the DD group compared to Controls, and a significant decrease in comparison to the FML group. FML is characterized by abnormal accumulation of SAT but not pain. It remains unclear whether the demonstrated differences are related to presence or absence of pain.

Although the palmitoleic/palmitic acid DI was not significantly decreased in DD subjects, the vaccenic/stearic acid DI was decreased. Vaccenic acid is actually derived through chain elongation of palmitoleic acid and is not made by direct desaturation of stearate [29]. The ratio of (palmitoleic + vaccenic)/palmitic acid was determined to better represent the product/precursor relationships of this pathway, and there was a decrease in this ratio in DD subjects compared to FML, and a trend toward a decrease in DD compared to obese Controls. This decrease suggests a dysregulation in desaturation in DD, in which the palmitic to palmitoleic acid desaturation pathway is relatively suppressed, leading to decreased elongation to vaccenic acid. These findings appear consistent with a previous report on two DD subjects that demonstrated decreased acetate-1-^14 ^C incorporation during MUFA synthesis in affected adipocytes compared to unaffected adipocytes in one subject, and decreased 18-carbon fatty acid production in the affected adipocytes of the second subject [25]. The new fatty acid production in the first subject, as measured by incorporation of ^14 ^C, was diminished in the 18:1 fatty acids, but the analysis did not distinguish between oleic and vaccenic acids. A more recent study [26] reported increased proportions of 18:1 fatty acid percent of total in 13 DD subjects in comparison to controls, but the control subjects were healthy and leaner with an average BMI of only 26 kg/m^2 ^while the DD subjects averaged 33.5 kg/m^2^. It is unknown how the DI of our study subjects would compare to those of non-obese controls or to unaffected adipose tissue from the same subjects. However, in many individuals with DD, MSL and FML, all SAT tissue is affected, making an intra-person comparison of SAT very difficult. Nevertheless, our data supports decreased desaturation in DD SAT compared to SAT of other obesity phenotypes.

We were unable to identify changes in SCD1 gene expression in our samples. Possible explanations for low gene expression include steady-state conditions, or low enzyme turnover. Altered protein expression and/or enzyme activity are potential causes of changes in the desaturation indicies in the absence of changes in gene expression. Discordant SCD1 mRNA and protein expression has been demonstrated in adipose tissue from obese humans [30]. These possibilities were not able to be explored within the scope of the present study and should be investigated in the future.

A lower desaturation index or a shift towards increased saturated fatty acids has been associated with inflammatory states. SCD1-deficient mice are protected against diet-induced obesity, however, they have increased plasma inflammatory markers and increased risk for atherosclerosis [31]. In a mouse model of colitis, fatty acid constituents of lysophosphatidylcholines were shifted towards increased saturated species relative to monounsaturated species, consistent with deceased SCD1 expression levels in the liver [4]. In patients with coronary artery disease, epicardial fat exhibits higher SFA, lower unsaturated fatty acids, and secretes more proinflammatory cytokines than SAT [5]. Induction of the adipocyte immune response by saturated fatty acids has been proposed to be mediated through the toll-like receptor-4/NFkB pathway [32]. In addition, adipose tissue can hypertrophy near areas of inflammation such as infected lymph nodes [33] or inflamed tissues [3]. Although obesity is associated with a low-grade, chronic inflammation in the adipose tissue, DD adipose tissue exhibits increased expression of IL-6 mRNA and evidence of pro-inflammatory macrophages, even in comparison to BMI- and weight-matched obese controls [23]. Decreased desaturation leads to decreased turnover of proinflammatory saturated fatty acids [34], and we see in our DD subjects an increased proportion of saturated myristic acid in comparison to Controls. A possible relationship between DD fatty acid metabolism and inflammation in our subjects should be further investigated as others found no evidence of increased inflammation in the DD SAT based on counting primarily perivascular immune cells in the DD SAT compared to controls [24]. An alternative explanation, that the lower DI in DD SAT reflects lower caloric intake cannot be dismissed but is less likely due to the presence of increased SAT in DD.

Additional contributing factors to inducing SAT growth other than lifestyle and inflammation have been reported. In mice with Prox1 haploinsufficiency, leakage of lymphatic fluid induced growth of fat [35]. Lymph can be a strong inducer of adipogenesis. It is interesting to note that manual lymphatic drainage decreased the amount of fat in a case of DD [36]. In a study on lipomas, increased lipoprotein lipase activity was implicated in the development of the abnormal adipose tissue masses [37].

With respect to PUFA, a low percent of total PUFA in MSL and a lower trend in DD were observed in comparison to obese controls. n-6 and n-3 PUFAs have been considered as anti-inflammatory fatty acids, with n-3 PUFAs having more potent anti-inflammatory effects. PUFA-deficiency may lead to consideration of whether inflammation is present in MSL. However, the DIs were not decreased in MSL. While LA trended lower in MSL, its downstream product AA trended higher than in DD, suggesting decreased AA turnover. AA is further metabolized to eicosanoids which can be either inflammatory or proinflammatory, so the significance of increased AA in MSL compared to DD is uncertain. An assessment of affected fat in MSL after intervention with dietary PUFAs (such as those found in fish oil) may be performed to determine the clinical significance of PUFA deficiency in MSL. In contrast, no clinical signs or studies in FML have suggested an inflammatory component in the development of the abnormal SAT in this RAD to date, and the cause for the growth of the hundreds of fatty nodules in this disorder remains unknown.

The relationships between desaturase activity and clinical markers of metabolic risk have yet to be determined. Although there were no differences that could be discerned in the correlations between clinical data and the palmitoleic/palmitic and oleic/stearic DI between groups, both DI were found to be correlated with the BMI and percent body fat in the total cohort. This is consistent with data showing that the DI in adipose tissue of obese mice are associated with BMI and the adiposity index [28]. The plasma DI has also been previously described as correlating to waist circumference in adults [7] and to the waist-hip ratio in adolescents [38]. Larger numbers of subjects in each of our study groups would be necessary to draw conclusions about whether any group differs in strength of association from the others, and to assess the influence of age and gender.

Although we were limited by availability of data on adiponectin, we found negative correlations between adiponectin levels and the oleic/stearic acid, vaccenic/stearic, and the (palmitoleic + vaccenic)/palmitic acid DI. An association between desaturase activity and adiponectin would be consistent with previously published data that demonstrated an inverse association between the plasma oleic/stearic DI and adiponectin in adolescent girls [8]. In Crohn's disease patients, mesenteric adipose tissue has been found to exhibit more saturated fatty acids [3,39], and a separate study showed increased adiponectin expression [40].

Our pilot study was limited by small subject numbers in the groups, lack of adipose tissue samples from a non-obese control group, and limited clinical and biochemical data. Subject numbers in the groups are small due to the rarity of these adipose disorders, and therefore the ability to examine correlations within groups is limited. Complete anthropometric data to assess degree of adiposity, as well as insulin resistance measures, would also have been desirable. Adipose tissue samples were also of insufficient quantity to perform protein expression studies or enzyme activity assays. Further studies are needed to explore the potential relationships between adipose tissue desaturase activity, inflammation, and other lipid parameters in people with and without RADs.

Increased SCD1 activity has been implicated not only in obesity, but also in the associated disorders of insulin resistance [41] and fatty liver [42]. Moreover, SCD1 activity may affect cell signaling processes through changes in the proportion of MUFA to SFA in cell membranes, and its role in cell proliferation and cancer is being investigated. Recent studies have implicated upregulation of SCD1 in lung cancer [43], prostate cancer [44], and breast cancer [45]. Pharmacologic inhibition of SCD1 has demonstrated a decrease in the MUFA/SFA ratio and cell proliferation in lung cancer cells [43], and has blocked signaling processes involved in oncogenesis and cancer progression in prostate cancer cells [44]. Therefore, SCD1 is emerging as an enzyme with diverse biological roles and requires further investigation.

## Conclusions

A lower desaturation index in adipose tissue from DD subjects compared to obese Control subjects and subjects with FML suggests differences in desaturase activity among the groups. What drives the difference in desaturase activity is unknown. A potential role of desaturase activity in affecting inflammation or lymphatic dysfunction in DD should be further investigated. The adipose tissue DIs positively correlate with BMI and percent body fat in this cohort of RAD and obese non-RAD subjects. Potential relationships between the DI and adipokines or adiposity measures in obesity and RADs should be explored.

## Methods

### Subjects and adipose tissue collection

Subjects were recruited at the General Clinical Research Center at the University of California, San Diego (UCSD) or the VA San Diego Healthcare System (VASDHS) in two separate research protocols. Subjects had a diagnosis of DD, MSL, or FML, or were newly diagnosed (by KLH) using published clinical criteria [9,16,46] after obtaining a history, performing an examination and obtaining a fat biopsy from affected adipose tissue. None of the subjects consumed fish oil supplements. Adipose tissue biopsy samples were obtained from affected SAT in DD, MSL and FML subjects and in matched areas in Control subjects using a 5 mm biopsy needle with intermittent suction as described previously [23]. We certify that all applicable institutional and governmental regulations concerning the ethical use of human volunteers were followed during this research. This study was approved by the Institutional Review Board at UCSD and the Research and Development Committee at the VASDHS. All subjects consented to their participation.

### Fatty acid extraction from adipose tissue

Samples of adipose tissue weighing 50 mg each were saponified overnight at 70°C with 100 μl of 30% KOH (w/v) and 100 μl of 200-proof ethanol. The samples were then acidified with HCl, and the fatty acids were extracted with petroleum ether three times and air dried. This method extracts total fatty acids, including all fatty acids from triglycerides, cholesteryl esters, phospholipids, and free fatty acids that may be present in the samples [47]. Fatty acids were derivatized as methyl esters with 0.5 N methanolic hydrochloric acid (Supelco, Bellefonte, PA), and dissolved in hexane for GC/MS analysis.

### Gas chromatography/mass spectrometry (GC/MS)

GC/MS analysis was carried out using a Hewlett-Packard model 5973 selective mass detector connected to a model 6890 gas chromatograph. 1 μl from each sample was injected per analysis, and all samples were analyzed three times. All fatty acids detected in each sample were separated on the gas chromatograph with a Bpx70 column (30-m length, 250-μm diameter, 0.25-μm film thickness) from SGE, Inc. (Austin, TX). The GC conditions were as follows: helium flow rate, 1 ml/min; initial oven temperature, 150°C, which was programmed to increase at 3°C/min to a final temperature of 221°C. The expected retention times for palmitate, palmitoleate, stearate, oleate, and vaccenate (for example) under these conditions were as follows: 6.6, 7.2, 9.5, 10.2, and 10.3 min, respectively. Mass spectra of fatty acids were acquired using electron impact ionization. Fatty acids were identified by comparison of retention time and mass spectra with known standards.

### Desaturation and elongation indices

Desaturation indices were determined by calculating the ratio of MUFA product to SFA precursor based on the relative intensities of the gas chromatogram peaks [7,8]. The area under each peak is proportional to the relative abundance of each fatty acid. The following desaturation indices were calculated: palmitoleic/palmitic, oleic/stearic, and vaccenic/stearic. In addition, the ratio of (palmitoleic + vaccenic)/palmitic was determined to more accurately represent the 16-carbon desaturation pathway since vaccenic is actually made from elongation of palmitoleic acid, and not directly from stearic acid. The elongation index was determined by calculating the saturated 18-carbon stearic/16-carbon palmitic ratio [1,29,38].

### Quantitative reverse transcription - PCR analysis

Total RNA was isolated from six subjects with DD, six subjects with FML (samples were insufficient in two subjects), five subjects with MSL and six obese Controls using TRIzol reagent (Invitrogen), according to manufacturers instructions. Up to 4 ug of total RNA was transcribed with Superscript III Synthesis Supermix kit (Invitrogen). Quantitative real-time PCR was performed using the Mx3000P Real-time PCR system (Stratagene) with SYBR Green dye (Molecular Probes) and Platinum taq polymerase (Stratagene). The primers used and their sequences are: SCD1 [NM_005063.4] (forward primer 5' cctagaagctgagaaactggtga 3' and reverse primer 5' acatcatcagcaagccaggt 3') and 18 s (forward primer 5' ggcctcactaaaccatccaa 3' and reverse primer 5'gcaattattccccatgaacg 3'). The levels of PCR product were calculated from standard curves established for each primer pair.

### Other clinical and biochemical measures

Because the samples and data were obtained through more than one study, complete data was not available (Table 5). Percent body fat was determined by dual X-ray absorptiometry scan measurements of fat-free mass and fat mass for whole body composition (Hologic Discovery W), and analysis using the software QDR DICOM for Windows XP. Serum cholesterol, triglyceride (TG), low-density lipoprotein (LDL), high-density lipoprotein (HDL), leptin, adiponectin, and non-esterified fatty acid (NEFA) levels had been determined using commercially available assay kits. Cholesterol and TG levels were measured by Trinder-based colorimetric end-point assays (Randox). LDL and HDL were determined directly by clearance methods (Randox). NEFA levels were quantified by an enzymatic colorimetric method using the Wako NEFA-HR2 kit. Adiponectin and leptin were measured by a multiplex immunoassay method using the Human Serum Adipokine Panels A, and B, respectively (Millipore).

### Statistical analyses

Data analyses were performed using GraphPad Prism 4.0 (GraphPad Software, Inc., San Diego, CA) and SigmaStat 3.5 (Systat Software, San Jose, CA). Differences between groups were examined with non-parametric two-sample Wilcoxon (Mann-Whitney) testing. Spearman's rank order correlations were performed using the desaturation indices and stearic/palmitic ratio against available clinical and biochemical data on the total cohort of subjects. The correlations against BMI and percent body fat were also made in males and females separately. All correlations were assessed qualitatively. The authors acknowledge that correlations made in groups of small subject numbers may demonstrate increased false-positive results. Mathematical adjustments for multiple comparisons were not done, however, 6 comparisons for each fatty acid parameter in Tables 2, 3, and 4 were made, and 83 correlations were performed.

## Competing interests

The authors have no competing interests and therefore have nothing to disclose.

## Authors' contributions

JKY performed GC/MS analysis on the adipose tissue fatty acids, Spearman's correlations, and drafted the manuscript. SAP carried out the RNA extraction, performed the PCR experiments, and contributed to manuscript preparation. KA participated in statistical analysis on the GC/MS fatty acid data and contributed to manuscript preparation. KLH recruited the study subjects, obtained the samples for analysis, provided oversight on the project, and contributed to manuscript preparation. All authors read and approved the final manuscript.
